# Outlining the skin-homing and circulating CLA^+^NK cells in patients with severe atopic dermatitis

**DOI:** 10.1038/s41598-024-53224-8

**Published:** 2024-02-01

**Authors:** Josenilson Feitosa de Lima, Franciane Mouradian Emidio Teixeira, Yasmim Álefe Leuzzi Ramos, Gabriel Costa de Carvalho, Anna Claudia Calvielli Castelo Branco, Naiura Vieira Pereira, Mírian Nacagami Sotto, Valéria Aoki, Maria Notomi Sato, Raquel Leao Orfali

**Affiliations:** Department of Dermatology, Laboratory of Dermatology and Immunodeficiencies (LIM-56), Faculdade de Medicina FMUSP, Universidade de Sao Paulo, Av. Dr. Eneas de Carvalho Aguiar, 255, 3o. andar ICHC, Sala 3016, Cerqueira Cesar, Sao Paulo, SP 05403-002 Brazil

**Keywords:** Cell biology, Immunology, Medical research, Pathogenesis

## Abstract

Atopic dermatitis (AD) is a complex, multifactorial skin disease, characterized by pruritus and predominant Th2 inflammation. Innate immune cells may play a role in AD development and are composed of granulocytes, macrophages, innate-like T cells, and innate lymphoid cells. This study investigates the phenotypic and functional profile of circulating CLA^+^ natural killer (NK) cells and its role in the skin-homing to NK cells infiltrated in adults’ skin with AD. We selected 44 AD patients and 27 non-AD volunteers for the study. The results showed increased frequencies of both CLA^+^CD56^bright^ and CLA^+^CD56^dim^ NK cell populations in the peripheral blood, mainly in severe AD patients. Upon SEB stimulation, we observed an augmented percentage of CLA^+^CD56^dim^ NK cells expressing CD107a, IFN-γ, IL-10, and TNF, reinforcing the role of staphylococcal enterotoxins in AD pathogenesis. Additionally, we demonstrated increased dermal expression of both NK cell markers NCAM-1/CD56 and pan-granzyme, corroborating the skin-homing, mostly in severe AD. Further studies are necessary to elucidate the potential role of NK cells in the chronification of the inflammatory process in AD skin, as well as their possible relationship with staphylococcal enterotoxins, and as practicable therapeutic targets.

## Introduction

Atopic dermatitis (AD) is a complex, multifactorial skin disease, characterized by pruritus and predominant Th2 inflammation^1^. The multifaceted imbalance involved in AD skin inflammation includes interactions between skin barrier dysfunction, adaptive immune inequity, genetic factors, altered epidermal microbiome, and innate immune cells (such as, innate lymphoid cells-ILCs, dendritic cells, mast cells, basophils, and eosinophils)^1–3^.

Innate immune cells are composed of granulocytes, macrophages, innate-like T cells, and three groups of ILCs; also, it has been suggested that they play a role in AD development^2,4^. Natural killer (NK) cells belong to type 1 ILCs, with cytotoxic activity, comprising 5–10% of the circulating peripheral blood mononuclear cells (PBMCs)^2,5^. They also include subsets with different repertoires, locations, functions, and developmental origins, and are present at a high frequency in the circulation, usually extravasating to tissues under inflammatory conditions, including AD^6^.

Concerning the innate immune system in AD, approximately 7–10% of AD patients develop skin viral infections, especially related to herpes simplex virus (eczema herpeticum-EH)^7^. A mice model study demonstrated a reduced NK cell activity, with decreased levels of granzyme B^+^NK cells, IFN-γ^+^NK cells, and perforin^+^NK cells, and no differences were detected in numbers of mature cytolytic NK cells between EH mice and healthy mice^7,8^. A recent study corroborated these data, showing that AD patients with EH also present reduced numbers of NK cells and a decrease in the ability to produce granzyme B under stimulation^7,9^.

NK cells may exert a role in immune homeostasis through endogenous mechanisms in the Th2 cells of AD patients^2,5^. Activated NK cells were described to be augmented in lesional AD skin biopsies, presenting as well decreased levels of peripheral blood NK cells with an altered composition of NK cell subsets, characterized by a reduction of mature CD56^dim^ CD57^+^ NK cells^2,5,10^. Notwithstanding, it has also been described small numbers of NK cells homing healthy skin, which indicates that further studies are necessary to elucidate the role of NK cells in AD, as well as how staphylococcal chronic colonization in AD skin influences this pathway^11^.

Cutaneous lymphocyte-associated antigen (CLA) is a glycoprotein with carbohydrate epitope expressed on the surface of different lymphocytes. CLA binds to E-selectin, expressed in endothelial cells and other tissues, during inflammation^12^. CLA^+^ T cells participate in skin homing and represent the main T cell population in AD^12^ and cutaneous lymphoma^13^ lesions. NK cells express CLA at levels comparable to T cells, and IL-2 and IL-12 stimulation increases CLA expression in NK and T cells^13^. Functional studies focusing on CLA^+^ T cells demonstrated that they are expressed in 15% of human circulating T cells, facilitating a differential migration of T cells to the skin, which indicates that they are functionally related to AD cutaneous inflammation^12^. In the dengue virus infection, peripheral blood NK cells showed CLA and other homing receptors (CCR5 and CXCR6) increased expression, in a pathway related to augmented plasmatic IL-18 levels, indicating an IL-18-dependent mechanism inducing NK cell proliferative response^14^. Our group has already demonstrated an increase in plasmatic IL-18 cytokine levels in AD patients^15^. However, the phenotypic and functional characteristics of CLA^+^ NK cells in AD are unknown.

Previous studies from our group revealed decreased PBMC proliferation response to staphylococcal enterotoxin A (SEA) and other recall antigens and mitogens, as well as a reduction of the polyfunctional response of T cells to SEA, a mechanism associated with the inhibition of the transcription factor Early Growth Response 2 (EGR2)^15–17^, suggesting possible anergy of T lymphocytes. Although CD4^+^ T cells depletion in AD^16,17^, we hypothesized that an active NK cell-mediated innate response may occur, perhaps to overcome the T cell’s anergic profile in response to staphylococcal enterotoxin B (SEB), which may reflect in AD cutaneous manifestations. In this study, we investigate the phenotypic and functional profile of circulating CLA^+^ NK cells and their role in the crosstalk with NK cells infiltrated in adults’ skin with AD.

## Results

### Skin-homing CLA expression on NK cells subsets in adult AD

**Figure 1:**
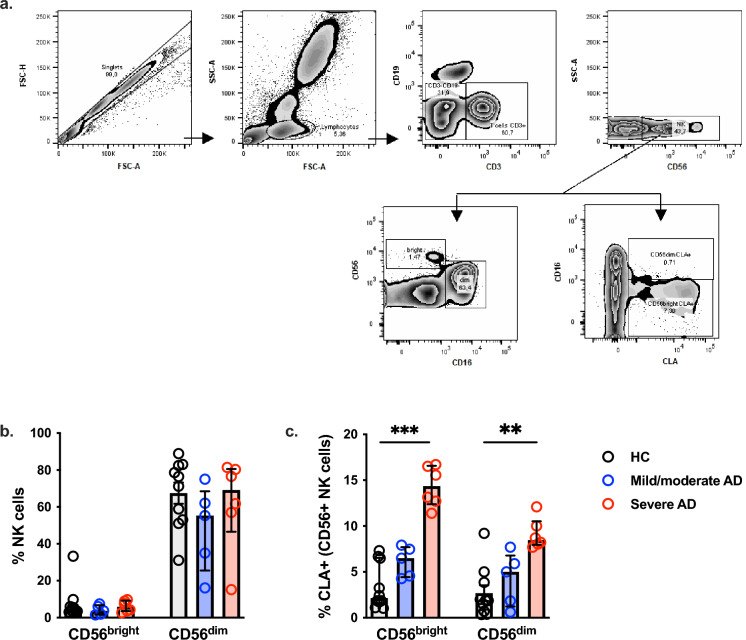
Phenotypic profile of circulating CLA^+^ NK cells in adult AD (ex vivo). (**a**) Representative gating strategy to analyze circulating CLA^+^ NK cells; (**b**) The frequency of CD56^bright^ and CD56^dim^ NK cells subsets (CD56^bright^CD16^−^ and CD56^dim^CD16^+^); (**c**) CLA expression on circulant CD56^+^ NK cells. HC (n = 10), mild/moderate AD (n = 5) and severe AD (n = 6). Data was plotted as a median with an interquartile range. *p < 0.05; **p < 0.01 and ***p < 0.001.

We assessed the expression of homing and activation molecules on the subtypes of NK cells in the peripheral blood of mild/moderate and severe AD patients. Figure 1a illustrates the NK cell analysis strategy, with our results showing no differences in the frequency of both CD56^bright^CD16^−^ and CD56^dim^CD16^+^ NK cell population comparing healthy controls (HC) and AD patients (Fig. 1b). On the other hand, a significant increase in the frequency of CLA expression was observed in subjects with severe AD, both in the CD56^bright^ (CD56^+^CD16^−^) and CD56^dim^ (CD56^+^CD16^+^) NK cell populations (Fig. 1c). This data suggests greater targeting of NK cells to the skin of individuals with severe AD.

### NK cell response in adult AD induced by in vitro stimulation

**Figure 2:**
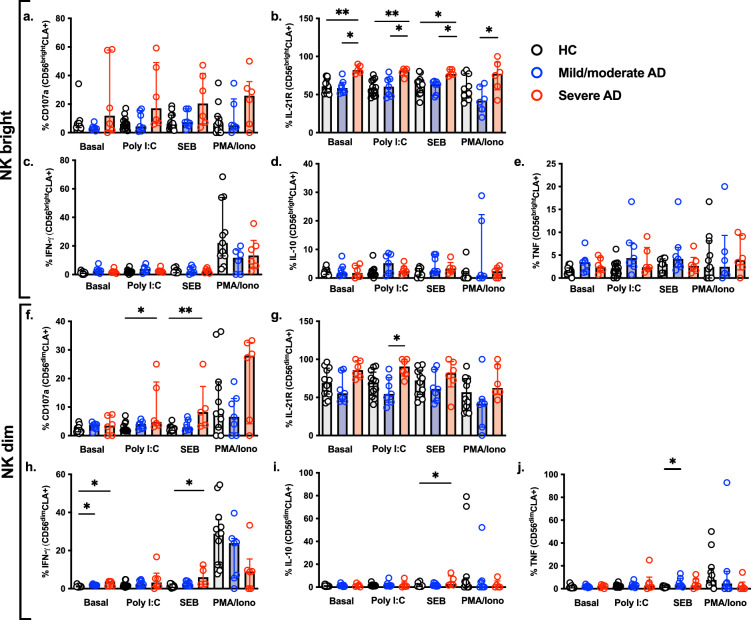
Effector molecules in CLA + NK subsets upon in vitro stimulation. PBMCs were stimulated with TLR3 agonist, SEB or PMA/Ionomycin for 20 h. The frequency of CD107a, IL-21R, IFN-γ, IL-10 and TNF was analyzed in NKCD56^bright^CD16- (**a**–**e**), NKCD56^dim^CD16^+^ (**f**–**j**) CLA^+^ cell populations. HC (n = 12), mild/moderate AD (n = 7) and severe AD (n = 6). Data was plotted as a median with an interquartile range. *p < 0.05 and **p < 0.01.

In this step, we evaluated the functional profile of NK cells subsets regarding CD107a, IL-21R, IFN-γ, IL-10, and TNF production, after stimulation with a toll like receptor (TLR) 3 agonist (Poly I:C—viral stimuli), SEB (bacterial stimuli), and PMA/Ionomycin (positive control). Supplementary Figure S1 illustrates the gating strategy for NK cells. We observed an increased expression of IL-21R in the CLA^+^ NK CD56^bright^ cell population at present in the baseline condition, which remained increased upon stimuli in severe AD patients in comparison to HC and mild/moderate AD groups (Fig. 2b). We did not detect differences in CD107a, IFN-γ, IL-10, and TNF in CLA^+^ NK CD56^bright^ cells (Fig. 2a, c, d, e). As for CLA^+^ NK CD56^dim^ cells, we observed that stimulation with SEB was the most significant method for inducing expression of CD107a, IFN-γ and IL-10 in severe AD patients (Fig. 2f, h, i). Similarly, SEB stimulated TNF expression in mild/moderate AD patients (Fig. 2j). The Poly I:C stimulation also increased the expression of CD107a and IL-21R in CLA^+^ NK CD56^dim^ cells, in patients with severe AD (Fig. 2f, g).

Noteworthy, in order to proper stimulate NK cells cytokine production, PMA/Iono was adopted as a positive control, which corroborates with previous studies in the literature^18,19^.

### NK expression profile in skin lesions from adults with AD

**Figure 3:**
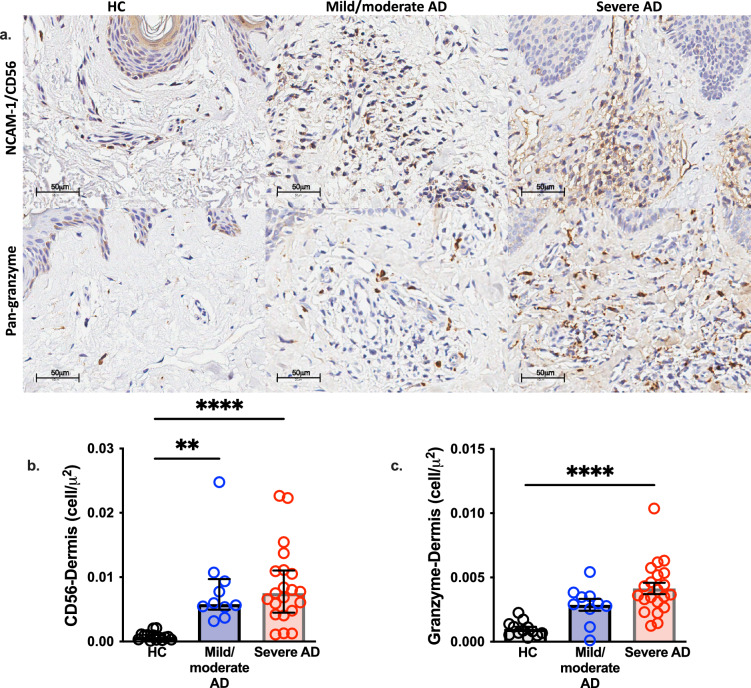
NK expression profile in skin lesions from adults with AD. Expression of NCAM-1/CD56 and granzyme in skin specimens from adults with AD assessed by immunohistochemistry. (**a**) Image of a skin specimen from a HC: NCAM-1/CD56 and pan-granzyme expression. NCAM-1/CD56 (**b**) and pan-granzyme (**c**) expression levels (cell/μm^2^) in the dermis of the control group (HC, n = 15) compared with mild/moderate AD (n = 10) and severe AD (n = 21) patients, evaluated by immunohistochemistry. Lines represent medians with interquartile ranges of NK markers in skin specimens. **p < 0.01 and ****p < 0.0001.

**Figure 4:**
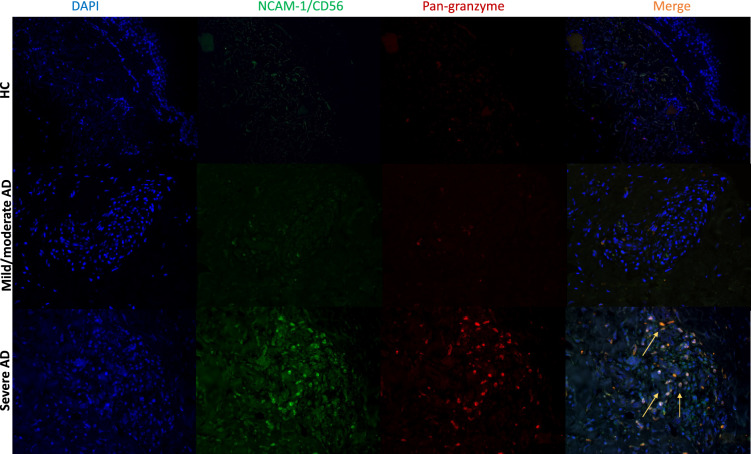
Immunofluorescence staining of NK cell markers in AD skin. Double-label immunofluorescence panels showing that dermal cells express both NCAM-1/CD56 (green) and granzyme NK cells (red). Yellow arrows show the merge of NCAM-1/CD56 and granzyme cells expressing NK cells.

Considering the CLA^+^ CD56 cells expression in the blood, we evaluate the NK cells migration to the skin through the expression of pan-granzyme and NCAM-1/CD56 (neuronal cell adhesion molecule-1), in AD skin compared to HC. Figure 3a shows the dermal expression profile from HC, mild/moderate AD, and severe AD individuals. We found an augmented expression of CD56 in both AD groups, and an increased expression of pan-granzyme in severe AD individuals compared to HC (Fig. 3b, c). We also evaluated NK cells assessing double-labeled cells expressing pan-granzyme and CD56 by immunofluorescence in skin sections of AD and HC samples. Figure 4 shows an increased expression of CD56^+^ cells co-expressing pan-granzyme at the dermis of severe AD patients.

## Discussion

Our study demonstrated an increased ex vivo expression of both CLA^+^ CD56^bright^ and CD56^dim^ NK cell population, mainly in severe AD patients, and an augmented functional ability to express IFN-γ, IL-10, and TNF under SEB stimulation in CLA^+^CD56^dim^ NK cells, reinforcing the role of staphylococcal enterotoxins in the AD pathogenesis. Additionally, we demonstrated an increased dermal expression of both NK cell markers NCAM-1/CD56 and pan-granzyme, corroborating the skin-homing mostly in severe AD.

When evaluating NK cells in CLA^+^ dependency, we noted an increased expression of NK CD56^bright^ and CD56^dim^ in severe AD. In fact, it has been described as an additive effect when CD56^dim^ NK cells were stimulated in vitro with a combination of IL-2 and IL-21 in peripheral blood from healthy donors^20^. In contrast, a study assessing patients with AD demonstrated that they have not only a global reduction in their blood NK cells but also a shift in subpopulations of NK cells, which is reflected by a loss of mature CD56^dim^ effector cells^5^. Other study revealed decreased frequencies of total CD56^+^ NK cells in AD supporting an increased NK cell apoptosis in the blood and extravasation in the inflamed skin^21^. In accordance with Mack et al.^5^, we believe that NK cells from AD patients may be undertaking an activation-induced cell death. More studies are needed to better elucidate the role of NK cells in the innate immune response of patients with AD^11^.

In the functional evaluation of NK cells, our study revealed increased expression of CD107a, IFN-γ, IL-10, and TNF under SEB stimulation in CLA^+^ NK^dim^ cells, reinforcing the role of staphylococcal enterotoxins in AD pathogenesis. Several studies have demonstrated that NK cell activation by staphylococcal enterotoxins increases IFN-γ^22,23^, granzyme, and perforin expression by NK cells, in a monocyte or T cell-dependent manner^24^. Poly I:C NK activation, viral ssRNA stimuli, elevated the expression of CD107a and IL-21R by CLA^+^ NK^dim^ cells in patients with severe AD, suggesting that antiviral cytotoxic response, mediated by TLR3 activation, is preserved in these patients^25^.

The increase in TNF expression at baseline in CD56^bright^ cells has already been evidenced in some inflammatory skin diseases (lichen planus, psoriasis, Sezary’s syndrome, and mycosis fungoides), as well as in our findings in adults with AD^26,27^. However, we did not observe similar effect in CLA + NK CD56^bright^ cell population. We detected an increased expression of IL-21R in the CLA^+^ NK CD56^bright^ cell population in the baseline condition, which remained increased upon stimuli in severe AD patients in comparison to HC and mild/moderate AD groups. It has been described that IL-21 exert effects on the expression of NK cell receptors, as well as increasing the expression of effector molecules, inducing perforin and granzyme B expression, and consequently increasing NK cell cytotoxicity in vitro^20,28^.

It is worth to mention that our study aimed to evaluate the functional response of CLA + NK cell subsets, which are not abundant in peripheral blood, but they still represent a relevant population for the understanding of AD pathogenesis.

To corroborate the NK cells skin-homing in AD lesions, we demonstrated that severe AD exhibited increased expression of pan-granzyme and NCAM-1/CD56 NK cell markers. Additionally, we found the expression of both NK cell markers in severe AD dermal specimens, in accordance with our previous findings. Previous studies described that granzyme-expressing cells are increased in lesions of patients with inflammatory skin diseases, such as AD, psoriasis, allergic contact dermatitis, and pityriasis rosea, compared with healthy skin, as well as plasma granzyme B levels being higher in patients with AD and psoriasis than in HC, with a positive correlation with Visual Analogue Scale (VAS) score. Our findings indicate that granzyme in severe AD cutaneous lesions, from NK cells, may exert a critical role in the initiation and perpetuation of itch and inflammation in AD, through the modulation of the innate response despite the adaptive response exhaustion^29^.

The potential role of NK cells in maintaining the inflammatory process in AD skin, as well as their possible relationship with staphylococcal enterotoxins and as practicable therapeutic targets, requires further studies.

## Methods

### Subjects

We enrolled 44 patients with AD (aged between 18 and 43 years; mean age: 28.93 ± 9.16; 24 males and 20 females) and 27 healthy non-AD volunteers (aged between 20 and 55 years; mean age: 36.48 ± 13.42; 8 males and 19 females) in this study. Thirteen AD patients and 12 healthy non-AD volunteers were selected for blood sample collection to be included in flow cytometry experiments, and 31 AD and 15 healthy non-AD volunteers skin samples were provided by our skin biorepository for immunohistochemistry experiments. AD was diagnosed according to the Hanifin & Rajka criteria^30^, and the disease severity was evaluated by the Eczema Area and Severity Index (EASI)^31^. AD patients were classified as mild/moderate (n = 17), or severe (n = 27), according to EASI^31^. None of the patients were under systemic treatment with oral steroids or immunosuppressants. The study was approved by the Ethics Committee of the University of Sao Paulo School of Medicine, and informed consent was obtained from all subjects. All methods were performed in accordance with the relevant guidelines and regulations of this institution. Table 1 summarizes the demographic data of the study.

**Table 1:** Demographic data of atopic dermatitis patients and healthy controls enrolled the study.

	Mild/moderate AD (n = 17)	Severe AD (n = 27)	HC (n = 27)
Age (years) mean ± SD	18–48 (27.9 ± 9.1)	19–61 (29.5 ± 9.3)	20–75 (36.4 ± 13.9)
Gender (M/F)	9M/8F	15M/12F	8M/19
EASI mean ± SD	3–27.2 (14.5 ± 6.5)	25–59.4 (40 ± 8.2)	NA

*AD* atopic dermatitis, *EASI* Eczema area and severity index (EASI scores: 0-clear or no eczema; 0.1 to 1.0-almost clear; 1.1. to 7-mild; 7.1 to 21-moderate; 21.1 to 50-severe; > 51-very severe), *SD* standard deviation, *HC* healthy controls, *M* male, *F* female, *NA* not applicable.

### Phenotypic characterization of NK cells by multiparametric flow cytometry

Whole blood was collected in EDTA-enriched tubes and stained with anti-CD3 (Q-dot 605; isotype Mouse BALB/c IgG1, κ; clone SK7), anti-CD19 (Horizon V-500; isotype Mouse IgG1, κ; clone HIB19), anti-CD56 (Alexa fluor 700; isotype Mouse IgG1, κ; clone B159), anti-CD16 (APC-Cy7; isotype Mouse BALB/c x DBA/2; clone 3G8) and anti-CLA (FITC; isotype Rat WI IgM, κ; clone HECA-452) (skin homing marker), for 20 min, then incubated for 15 min with FACS lysing solution (BD FACS lysing; BD) to lyse the erythrocytes, and washed in isotonic solution (Hemoton SPEC, Sao Paulo, Brazil). All antibodies were purchased from BD Biosciences, San Jose, CA, USA. A total of 200,000 events were acquired and further analyzed by flow cytometry (LSR Fortessa, BD Biosciences). NK cells were identified by gating cells that were positive for CD56 and excluding CD3^+^ and CD19^+^ cells, and CD56^bright^ and CD56^dim^ NK cells were identified and analyzed as described elsewhere^32^. We performed fluorescence minus one (FMO) control for all antibody panels to check for proper compensation and to define positive signals. Data analysis was performed using FlowJo v10 software (Tree Star, Ashland, OR, USA).

### Functional characterization of NK cells by multiparametric flow cytometry

PBMC (2 × 10^6^/well) were isolated by Ficoll-Hypaque density gradient (GE Health Care, Uppsala, Sweden) centrifugation and cultivated in 96-well microplates (Costar) at 37 °C and 5% CO2, as described elsewhere^17,18,32^, in the presence of SEB (1.0 μg/mL; Sigma-Aldrich, St. Louis, MO, USA), TLR3 agonist (polyinosinic-polycytidylic acid—Poly(I:C); 20 μg/mL; InvivoGen, San Diego, CA, USA) and PMA (Phorbol 12-Myristate 13-Acetate—10 ng/mL; Sigma)/Ionomycin (1 µg/mL; Sigma). The anti-CD107a (PerCP-Cy5-5; isotype Mouse BALB/c IgG1, κ; clone H4A3) antibody was added to the cells culture to detect degranulation of NK cells. The addition of Brefeldin A (10 μg/mL; Sigma) occurred 4 h after the start of cultivation and incubated at 37 °C at 5% CO2 for 20 h. Next, the cells were stained with the viability marker LIVE/DEAD PE-Texas Red (Invitrogen, Carlsbad, CA, USA) for 30 min at room temperature. Then we incubated cells with Cytofix/Cytoperm™ kit (cat. 554714, BD) according to manufacturer’s recommendation and stained them with monoclonal antibodies for anti-CD3 (Qdot 605), anti-CD56 (Alexa Fluor 700), anti-CD16 (APC-Cy7), anti-CLA (FITC) (skin-homing marker), and anti-IL-21R (APC). The intracellular staining was performed with monoclonal antibodies conjugated with fluorochromes anti-IL-10 (PE; isotype Rat IgG2a, κ; clone JES3-19F1), anti-IFN-γ (Horizon V450; isotype Mouse IgG1, κ; clone B27), and anti-TNF (PE-Cy7; isotype Mouse IgG1, κ; clone MAb11). All antibodies were purchased from BD Biosciences. Cells were fixed with 1% paraformaldehyde, and 250,000 events were acquired on an LSR Fortessa flow cytometer (BD). We performed FMO control for all antibody panels to check for proper compensation and to define positive signals. FlowJo v10 (Tree Star) was the chosen software for data analysis.

### Immunohistochemistry of NK cells

Immunohistochemistry was performed on slices with a 4 µm thickness of paraffin-embedded samples, as already described^33–35^. The primary antibody Pan-granzyme (isotype Goat polyclonal IgG, SC-1969; Santa Cruz Biotechnology, Dallas, TX, USA) was utilized at 1:50 dilution, and the detection system was LSAB + System-HRP (K0690; DakoCytomation, Carpinteria, CA, USA); NCAM-1/CD56 (isotype Mouse monoclonal IgG1, ab200698; Abcam, Cambridge, MA, USA) was utilized at 1:100 dilution, and the detection system was Novolink Max Polymer Detection System (RE7280-K; Leica Biosystems, Newcastle Upon Tine, UK), and the chromogen used was DAB (3,3′ diaminobenzidine, D5637, Sigma). We performed scanning of immunohistochemically stained specimens using Aperio Scan-scope Cs (Aperio Technologies, Vista, CA, USA). Images of the scanned stained specimens were analyzed utilizing the software Image-Pro Plus version 4.5.0.29 (Media Cybernetics Inc., Bethesda, Maryland, USA)^34,36^. Using the software, we obtained the calculation of the total tissue distribution of pan-granzyme and NCAM-1/CD56 dividing the total number of doubly stained cells by the total area measured in the dermis. The expression of the measured markers was normalized by the total area and expressed in cells/μm^2^.

### Immunofluorescence assay

The immunofluorescence was performed on slices with a 4 µm thickness of paraffin-embedded samples, as already described^33^. After dewaxing, samples were fixed with paraformaldehyde 4% and blocked with phosphate-buffered saline with bovine serum albumin (PBS-BSA) 2% for 30 min. Incubation of the primary antibodies Pan-granzyme (isotype Goat polyclonal IgG, SC-1969, Santa Cruz Biotechnology) and NCAM-1/CD56 (isotype Mouse monoclonal IgG1, ab200698, Abcam) went on overnight at 4 °C. After incubation, permeabilization was performed using Triton X-100 surfactant (Sigma), followed by the application of diamidino phenylindole (DAPI, D1306; ThermoFisher Scientific, Waltham, MA, USA) for nuclear identification, and rabbit anti-goat Alexa 488 fluorescein (A11008, ThermoFisher Scientific) (for NCAM-1/CD56 staining) and donkey anti-mouse Alexa 555 fluorescein (A31570, ThermoFisher Scientific) (for Pan-granzyme staining) secondary antibodies were diluted and incubated for 90 min at room temperature. Images were acquired utilizing the appropriate filters of an Axio Imager A1 inverted immunofluorescence microscope (Zeiss, Oberkochen, Germany).

### Statistical analysis

We analyzed the comparison of 2 groups using the non-parametric Mann–Whitney test and the comparison of 3 groups using the Kruskal–Wallis test, with Dunn’s post-test (statistically significant when *p* < 0.05).

### Supplementary Information

Supplementary Information.

## Data Availability

All data generated or analyzed during this study are included in this published article [and its supplementary information files].
