# Supplementary material for: Outlining the skin-homing and circulating CLA+NK cells in patients with severe atopic dermatitis
Source: Sci Rep. 2024 Feb 1;14:2663. doi: 10.1038/s41598-024-53224-8 (PMC10834414; doi:10.1038/s41598-024-53224-8)
Supplement: Supplementary file 1 — Supplementary Information. [file 41598_2024_53224_MOESM1_ESM.docx]

**Supplementary Figure 1**


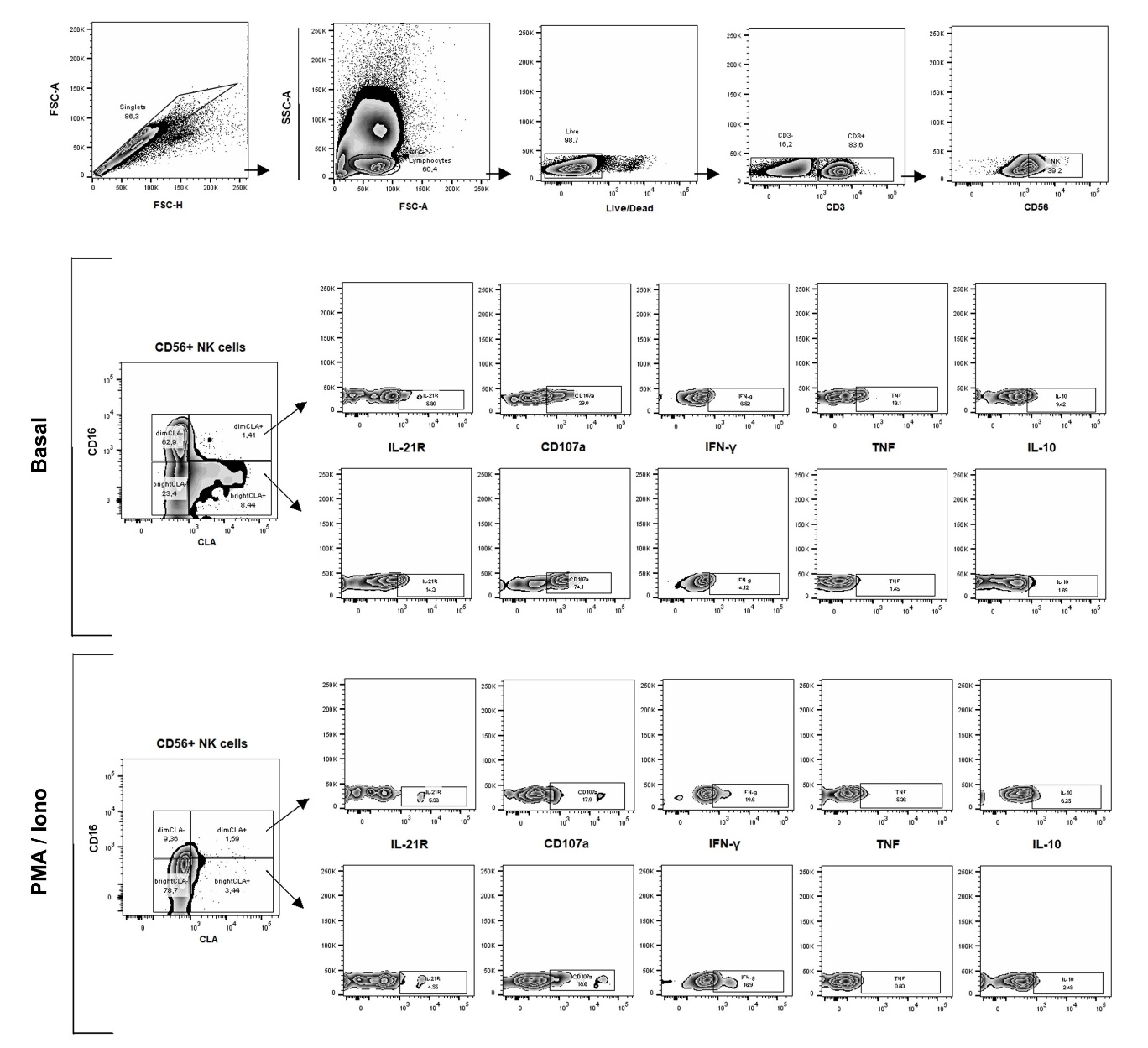


**Supplementary Figure S1:** Representative flow cytometry gating strategy analysis of CLA+ NK cells subsets under *in vitro* stimulation. Live lymphocytes were derived from single cells, followed by CD3- and CD56+ expression to characterize NK cell population. CD56+ NK cells were selected according to the expression of CLA and CD16 and divided into NK dimCLA+ (CD56+CD16+CLA+) and brightCLA+ (CD56+CD16-CLA+). The expression of IL-21R, CD107a, IFN-γ, TNF and IL-10 was evaluated into those NK cell subsets. The data represents basal (unstimulated) and PMA / Ionomycin (positive control) conditions from a severe AD patient.
